# Cone beam computed tomography (CBCT) in periodontal diseases: a Systematic review based on the efficacy model

**DOI:** 10.1186/s12903-020-01106-6

**Published:** 2020-07-08

**Authors:** Hassan Assiri, Ali Azhar Dawasaz, Ahmad Alahmari, Zuhair Asiri

**Affiliations:** 1 Department of Oral Biology and Diagnostic Science, King Khalid University, College of Dentistry, Abha, Saudi Arabia; 2 Department of Periodontology, King Khalid University, College of Dentistry, Abha, Saudi Arabia; 3 College of Dentistry, King Khalid University, Abha, Saudi Arabia

**Keywords:** Cone-beam computed tomography, Digital volume tomography, Furcation defects, Infra-bony defects, Periodontitis

## Abstract

**Background:**

Periodontal diseases are prevalent among adult populations. Its diagnosis depends mainly on clinical findings supported by radiographic examinations. In previous decades, cone beam computed tomography has been introduced to the dental field. The aim of this study was to address the diagnostic efficacy of cone-beam computed tomographic (CBCT) imaging in periodontics based on a systematic search and analysis of the literature using the hierarchical efficacy model.

**Methods:**

A systematic search of electronic databases such as PubMed, Scopus, Web of Science, and Cochrane was conducted in February 2019 to identify studies addressing the efficacy of CBCT imaging in Periodontics. The identified studies were subjected to pre-identified inclusion criteria followed by an analysis using a hierarchical model of efficacy (model) designed for an appraisal of the literature on diagnostic imaging modality. Four examiners performed the eligibility and quality assessment of relevant studies and consensus was reached in cases where disagreement occurred.

**Results:**

The search resulted in 64 studies. Of these, 34 publications were allocated to the relevant level of efficacy and quality assessments wherever applicable. The overall diagnostic accuracy of the included studies showed a low or moderate risk of bias and applicability concerns in the use of CBCT. In addition, CBCT is accurate in identifying periodontal defects when compared to other modalities. The studies on the level of patient outcomes agreed that CBCT is a reliable tool for the assessment of outcomes after the treatment of periodontal defects.

**Conclusion:**

CBCT was found to be beneficial and accurate in cases of infra-bony defects and furcation involvements.

## Background

Periodontal diseases affect the structures surrounding the teeth [1–3]. They range from the mildest form of gingivitis to the most aggressive form of periodontitis. Gingivitis is limited to the inflammation of gingiva without deep involvement of teeth-supporting structures such as the alveolar bone. On the other hand, periodontitis does extend to the alveolar bone [4–7]. It starts with the formation of a periodontal pocket and, consequently, if not treated, leads to bone and tooth loss. Another manifestation of the periodontal diseases in molar-premolar teeth is the formation of furcation defects [8–11]. Since gingivitis affects only the soft tissue, its diagnosis and treatment rely solely on clinical findings including redness, puffiness, and bleeding [12–14]. However, periodontitis could lead to bone resorption depending on its severity; hence, its diagnosis and treatment planning relies on clinical methods supported by radiographic imaging [15–17].

There are several risks to using clinical examination alone, which could prevent the accurate diagnosis of periodontitis, including gingival tissue consistency, inflammation severity, pressure while probing, probe size, probing angulation, and dental restoration existence [18, 19]. In dental practice, practitioners routinely utilize conventional radiography such as periapical, bitewing, and panoramic x-ray to evaluate the bone loss and overall condition of the periodontal disease [18]. Nevertheless, the two-dimensional x-ray has some limitations, mainly due to the overlapping of structures [20]. Thus, the detection of bone craters, inter-radicular bone loss, and lingual and buccal marginal bone loss necessitate the consideration of three-dimensional radiography [17, 21–24].

Cone-beam computed tomography (CBCT) has been used frequently in the last two decades in dentomaxillofacial region [25]. It has many advantages compared to conventional computed tomography (CT) including low price, low radiation dose, and ease of accommodation at dental offices [25–27]. In addition, it has the ability to view the structures in three dimensions [28–30]. CBCT images of periodontal bone lesions offer a highly informative value. The spatial representation of the alveolar bone in all three planes has a significant role in periodontology, as treatment decisions and long-term prognosis rely on it [11]. Accordingly, it can play a potential role as an adjunct to clinical examination in the case of periodontal diseases [28, 31, 32].

Evidence-based dentistry aims to identify the best available evidence to justify the efficacy and use of any dental imaging or test in actual practice. Accordingly, Fryback and Thornbury came up with a hierarchal model of efficacy in the early nineties to sort out the best available evidence for a diagnostic tool [33].

There are several published studies on the role of CBCT in periodontal diseases in the literature [13–15].

However, the extent to which CBCT is efficient and accurate in the diagnosis, treatment planning, decision-making, and treatment outcomes of periodontal diseases remains ambiguous. On the path to routine use, especially under consideration of higher radiation exposure to patients, the gain in additional information of clinical relevance has to be explored and evaluated. Consequently, we conducted a systematic review to address the efficacy of CBCT in periodontal diseases.

## Methods

This review was conducted based on guidelines from Preferred Reporting Items for Systematic Review and Meta-Analysis (PRISMA) [34] and guidance from the center for reviews and dissemination (CRD) for undertaking a systematic review in health care [35]. The eligibility criteria for inclusion and exclusion were set. Then, the included studies were assigned to the suitable level of efficacy. In the meantime, the review question was designed according to the PICO (**P**opulation, or **P**roblem, **I**ntervention or Exposure, **C**omparison, **O**utcome) element [36]. Finally, each study was evaluated for quality using the predetermined tool for quality assessment (QUADAS 2).

### Criteria for inclusion

I. Original studies
II. Systematic reviews
III. The study must assess the role of CBCT in plaque-induced periodontal disease
IV. Each study can be on any level of the efficacy model [33]
V. Studies addressing CBCT accuracy should compare it to clinical or radiographic measurements

### Criteria for exclusion

I. Case reports
II. Narrative reviews
III. Languages other than English
IV. Studies addressing periapical periodontitis caused by pulpal infection
V. Studies addressing the bone status for the purpose of dental implant
VI. Studies highlighting the use of CBCT to address artificially created bone defects

- **Problem specification:**

The research question was defined as “what is the diagnostic efficacy of CBCT in individuals with periodontal diseases?”

- Literature search:

Four databases PubMed, Scopus, Cochrane, and Web of Science were searched till February 2019 to identify the relevant studies. The search strategy is shown in Table 1.

- Study retrieval:

**Table 1:** Study search strategy

Database and its supplies	Index terms	Results
Pubmed (US National Library of Medicine (NLM))	Cone beam computed tomography [MeSH]) OR Cone beam computed tomography [Title/Abstract]) OR Cone beam CT [Title/Abstract]) OR CBCT [Title/Abstract]) OR Dental computed tomography [Title/Abstract]) OR Digital volume tomography [Title/Abstract]) OR Volumetric tomography [Title/Abstract]) OR 3D image [Title/Abstract]) OR Three dimensional imaging [Title/Abstract]) OR Imaging, three dimensional [MeSH] AND Periodontitis [MeSH] OR Periodontal disease [Title/Abstract]) OR Periodontal diseases [Title/Abstract]) OR Furcation defects [MeSH]) OR Furcation involvement [Title/Abstract]) OR Alveolar bone loss [MeSH]) OR Intrabony defect [Title/Abstract]	539
Scopus (Elsevier)	TITLE-ABS-KEY (Cone beam computed tomography) or TITLE-ABS-KEY (Cone beam computed tomography) or TITLE-ABS-KEY (Cone beam CT) or TITLE-ABS-KEY (CBCT) or TITLE-ABS-KEY (Dental computed tomography) or TITLE-ABS-KEY (Digital volume tomography) or TITLE-ABS-KEY (Volumetric tomography) or TITLE-ABS-KEY(3D image) or TITLE-ABS-KEY (Three-dimensional imaging) AND TITLE-ABS-KEY (Periodontitis) or TITLE-ABS-KEY (Periodontal disease) or TITLE-ABS-KEY (Periodontal diseases) or TITLE-ABS-KEY (Furcation involvement) or TITLE-ABS-KEY (Furcation defects) or TITLE-ABS-KEY (Intrabony defect)	746
Cochrane (Wiley InterScience)	Cone beam computed tomography [MeSH] or Cone beam computed tomography (word variations ti, ab, kw) or Cone beam CT (word variations ti, ab, kw) or CBCT (word variations ti, ab, kw) or Dental computed tomography (word variations ti, ab, kw) or Digital volume tomography (word variations ti, ab, kw) or Volumetric tomography (word variations ti, ab, kw) or 3D image (word variations ti, ab, kw) or Iamging, three dimensional [MeSH] or AND Periodontitis [MeSH] or Periodontal disease (word variations ti, ab, kw) or Periodontal diseases (word variations ti, ab, kw) or Furcation involvement (word variations ti, ab, kw) or Furcation defects [MeSH] or Alveolar bone loss [MeSH] or Intrabony defect (word variations ti, ab, kw)	71
Web of Science	Cone beam computed tomography (Topic) or Cone beam CT (Topic) or CBCT (Topic) or Dental computed tomography (Topic) or Digital volume tomography (Topic) or Volumetric tomography (Topic) or 3D image (Topic) or Three dimensional imaging (Topic) or AND Periodontitis (Topic) or Periodontal disease (Topic) or Periodontal diseases (Topic) or Furcation defects (Topic) or Furcation involvement (Topic) or Alveolar bone loss (Topic) or Intrabony defect (Topic)	555

The resultant studies were subjected to a duplicate check on the RefWorks database. The studies were then reviewed by four authors for relevance based on inclusion and exclusion criteria. After that, the studies meeting the eligibility criteria were assigned for full-text screening. Where uncertainty was present, discussions were conducted between the authors to reach an agreement on whether to include or exclude a study based on the predefined inclusion and exclusion criteria.

- Data extraction & quality assessment:
  Finally, each of the selected studies was assigned for data extraction and analysis. After that, each study was allocated its suitable level of efficacy. A Revised Tool for the Quality Assessment of Diagnostic Accuracy Studies (QUADAS 2) was used for quality assessment. This tool contains four domains: patient selection, index test, reference standard, and flow and timing. Each domain is assessed in terms of risk of bias and the first three domains are assessed in terms of concerns regarding applicability. Signaling questions are included to help judge the risk of bias [37].

## Result

### Studies allocation

The search strategy of the four databases yielded 1717 articles: PubMed 539, Scopus 746, Cochrane 71, and Web of Science 555. After a duplicate check using RefWorks, the result came up to 1262. These were subjected to the title and abstract screening by the two authors. A set of 65 studies were linked to the full-text review. A total of 28 articles were excluded because they did not possess at least one of the inclusion criteria. Studies reported by [28, 38–50] were ex vivo studies and out of our review.

**Fig. 1:**
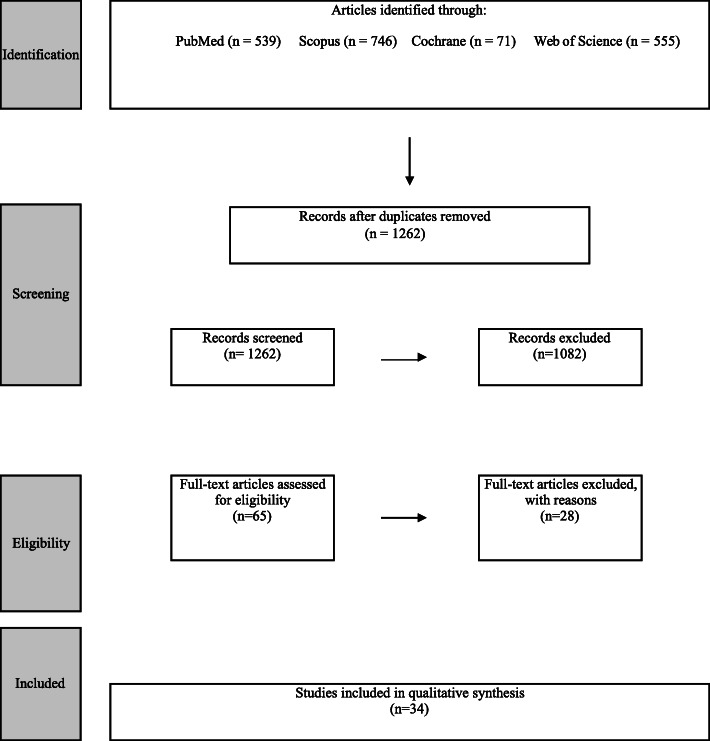
The results for systematic reviews according to the PRISMA flow chart

Plaque-induced periodontitis was not addressed, therefore, studies on that issue were excluded. In addition, studies that did not belong to any level of efficacy were disregarded [51–55]. Studies that addressed bone density conducted by Al Zahrani et al. [56] and bone coverage conducted by Ferriera et al. [57] were also excluded. Published studies by Evangelista et al. [58], Sun et al. [59], and Leung et al. [60] discussed only the naturally occurring dehiscence and fenestration, hence, they were disregarded. Studies reported by Goodarzy et al. [61] and Nagao et al. [62] were excluded because they did not include patients having periodontitis. The case report presented by Naitoh et al. [63] was disregarded as well. Studies published in languages other than English; reported by Deng et al. [64]) was excluded. Figure 1 shows the results for systematic reviews according to the PRISMA flow chart. Table 2 shows the studies that were included and their suitable efficacy level.

- Quality assessment
  After allocating each study its suitable efficacy level, special tools of quality assessment were used for each one as described in the literature [37].
- Technical efficacy studies:
  There was no study identified on this level of efficacy.
- **Diagnostic accuracy studies**:
  The results revealed eighteen studies [65, 69, 71, 74, 76, 81, 84, 86–96] on diagnostic accuracy. The QUADAS-2 assessment tool was used for quality assessment [34]. Table 3 reveals the results of the quality assessment using QUADAS-2.
  There were three studies that included a previously published systematic, manual search of the reference lists of the included articles [64, 81, 87], among which one study by Deng et al. [64] was found to be published in a Chinese language and hence excluded.
- **Diagnostic thinking efficacy**:
  Only one study was found to be on the level of diagnostic thinking efficacy [66]. The author investigated the effect of CBCT on the treatment decision-making after taking into consideration the clinical parameters.
- **Therapeutic efficacy**:
  In this level of efficacy, only one study, Pajnigara et al. [67], seemed relevant.
- **Patient outcome efficacy**:
  Our research resulted in eight studies in which CBCT was used to address the patients’ outcomes in relation to periodontal disease. All of the studies are randomized clinical trials [68, 72, 75, 77, 79, 82, 85]. Table 4, the CASP (critical appraisal skills program) checklist, was used to assess outcomes.
- **Societal efficacy**:
  Only one study was found to be relevant in this level of efficacy, Walter et al. [69]. The quality assessment was done using the QUADAS 2 tool.

**Table 2:** Studies that were included and their suitable efficacy level

Included studies							Excluded studies	Exclusion reason
Efficacy levels						Systematic Review		
1	2	3	4	5	6			
	Nagao et al. 2006 [65]	Walter et al. 2009 [66]	Pajnigara et al. 2016 [67]	Grimrad et al.2009 [68]	Walter et al. 2012 [69]	Walter et al.2016 [70]	Micsh et al. 2006 [42]	Ex vivo
	Walter et al. 2010 [71]			Gupta SJ et al. 2014 [72]		Anter et al.2016 [73]	Naitoh et al. 2006 [63]	Case report
	De Faria et al.2012 [74]			Khosropana et al.2015 [75]		Nikolic-Jakoba et al.2016 [6]	Nagao et al.2007 [62]	Did no check for periodontitis
	Fiejo et al. 2012 [76]			Bhavsar et al. 2016 [77]		Haas et al. 2018 [78]	Vandenberghe et al. 2007 [47]	Ex vivo
	Walter et al. 2012 [69]			Pajnigara et al. 2017 [79]		Choi et al. 2018 [80]	Mol et al. 2008 [43]	Ex vivo
	Raichur et al. 2012 [81]			Dutra et al. 2017 [82]		Woelber et al. 2018 [83]	Vandenberghe et al. 2008 [46]	Ex vivo
	Marinescu et al. 2013 [84]			Nemoto et al. 2018 [85]			Noujeim et al. 2009 [44]	Ex vivo
	Qiao et al. 2013 [86]						Leung et al. 2010 [60]	Ex vivo
	Haghgoo et al.2014 [87]						Evangelista et al.2010 [58]	Addressed naturally occurring dehiscence and fenestration in patients with malocclusions
	Banodkar et al. 2015 [88]						Ferreira et al.2013 [57]	Study targets bone coverage
	Cimbaljevic et al. 2015 [89]						DG Pour et al. 2015 [61]	Does not involve patients with periodontitis
	Darby et al. 2015 [90]						AlShaer et al. 2013 [55]	Does not belong to any level of efficacy
	Li F et al. 2015 [91]						Fliener et al. 2013 [40]	Ex vivo
	Guo et al. 2016 [92]						Kamuroglu et al. 2013 [41]	Ex vivo
	Zhu et al. 2016 [93]						Amorfini et al.2014 [54]	Does not belong to any level of efficacy
	Suphanantachat et al. 2017 [94]						Sun L et al. 2015 [59]	Excluded patients with periodontal diseases
	Padmanabhan et al. 2017 [95]						Yang et al. 2015 [53]	Does not belong to any level of efficacy
	Zhang et al. 2018 [96]						Bagis et al. 2015 [38]	Ex vivo
							Takeshita et al. 2015 [28]	Ex vivo
							Kolsuz et al. 2015 [48]	Ex vivo
							Kamburoğlu et al. 2015 [49]	Animal study and not plaque-induced periodontitis
							Deng et al. 2015 [64]	Not in English Language
							Lim et al. 2016 [51]	Does not belong to any level of efficacy
							Al-Zahrani et al. 2017 [56]	Targets bone density in aggressive periodontitis and normal patients
							Ozcan et al.2017 [52]	Does not belong to any level of efficacy
							Almeida et al.2017 [39]	Ex vivo
							Salineiro et al.2017 [45]	Ex vivo
							Elashiry et al. 2018 [50]	Not plaque-induced periodontitis

**Table 3:** Quality assessment of diagnostic accuracy studies using QUADAS2 tool

Study	Sample	Index test	Reference standard (comparator)	Risk of bias				Applicability concern		
				Patient Selection	Index test	Reference test	Flow and timing	Patient selection	Index test	Reference standard
Nagao et al. 2006 [65]	Developing novel method for alveolar bone resorption assessment caused by periodontitis	Dental CT	Not applicable			N/A				N/A
Walter et al. 2010 [71]	14 patients, five women and nine men, with a mean age of 57.0 years (range 42–81 years) and a diagnosis of generalized chronic periodontitis	CBCT Accuitomo with settings in the range of 74–90 kV, 5–8 mA and voxel sizes in the range of 0.08–0.25 mm	Intrasurgical measurements							
De Faria et al. 2012 [74]	Image records of 39 teeth from 11 adult patients of both genders, aged between 39 and 66 years	CBCT i-CAT with 120 kV and 36.12 mAs. The field of view (FOV) was 6 cm and the voxel size was 0.260.260.2 mm | Intraoral radiography (Kodak) with 120 kV and 36.12 mAs. The field of view (FOV) was 6 cm and the voxel size was 0.260.260.2 mm	Not available				N/A			N/A
Fiejo et al. 2012 [76]	6 patients with 12 measurement sites	CBCT i-CAT with voxel size of 0.2 mm and 40 seconds acquisition time	Intrasurgical measurements							
Walter et al. 2012 [69]	12 patients, 3 women and 9 men, with an average age of 57.5 years (range: 41–80 years) and a diagnosis of generalized chronic periodontit-is	CBCT 3D Accuotom with volumes of 4 9 4 cm to 6 9 6 cm	Intrasurgical measurements							
Raichur et al. 2012 [81]	7 patient (3 males and 4 females) having moderate to severe periodontitis	Digital volume tomography Kodak 9000 C3D with (exposure parameters were set at 70-74 kV, 10 mA and 10.8 seconds} & Radiovisiography (with a size #2 charged couple device (CCD) intraoral digital sensor§ and a standard X-ray unit* operating at 60-63 kV, 8 mA and 0.25-0.32 sec)	Direct measurements with UNC 15 probe							
Marinescu et al. 2013 [84]	19 patients with (presenting a total of 25 lower molars with different degrees of furcation defects )	CBCT	Clinical measurements							
Qiao et al. 2013 [86]	15 patients (9 women and 6 men) with an average age of 43.5 years and a diagnosis of generalized chronic periodontitis	CBCT Accuitomo with settings in the range 74–90 kV and 5–8 mA and voxel size of 0.125 × 0.125 × 0.125 mm	Intrasurgi-cal measurem-ents							
Haghgoo et al. 2014 [87]	50 interproximal sites in patients having periodontitis are assessed	CBCT (Newtom 3G, Verona. Italy) and direct digital intraoral radiography (Sopro-La Ciotat-France) was taken.	Intrasurgi-cal findings							
Banodkar et al. 2015 [88]	15 patients with hundred periodontal bone defects	CBCT promax (Planmec) with 90 kv, voxel size-400 μm, current-10 mA, and exposure time-13 s	Intrasurgical measurements							
Cimbaljevic et al 2015 [89]	15 patients (4 men and 11 women aged 35 to 60 years; mean age 44.5 ± 8.4 years) with 174 furcation sites	CBCT unit (SCANORA 3Dx, Soredex) with 80 × 100 mm field of view, 0.25 mm voxel size, 90 kV tube voltage, 10 mA tube current, and 2.4 seconds active scanning time | Probing using a Nabers probe (PQ2N, Hu-Friedy)	Not available			N/A				N/A
Darby et al. 2015 [90]	Clinical records ( Retrospective ) from the Periodontics clinic at the Royal Dental Hospital of Melbourne (RDHM)	CBCT i-CAT with slice thickness 1 mm, voxel size 0.2 mm. 120 kV, 20.7 mAs^−1^, 14.7 s acquisition time)	Not available			N/A				N/A
Li F et al. 2015 [91]	44 patients (22 men and 22 women) with 44 intrabony defects	CBCT New Tom, Verona, Italy (12-17mA and 110 Kv), and digitalperiapical radiography (70kVp and 12-25mA)	Intrasurgi-cal measurements made with a probe (HU-Friedy)							
Guo et al. 2016 [92]	6 patients (2 males and 4 females)	CBCT 3D Accuitomo with a field of view of 4 3 4 cm, tube voltage of 75–85 kVp and tube current of 5 mA. The voxel size used was 0.125 3 0.125 3 0.125 mm.	Intrasurgical measurem-ents							
Zhu et al. 2016 [96]	11 patients (Thirty-nine sites with degree II FI, classified by probing of 21 maxillary molars, were investigated}	CBCT 3D Accuitomo with volumes of 4 × 4 to 6 × 6 cm, with a setting in the range of 80 kV, 5.0 to 6.3 mA and a voxel size of 0.125 × 0.125 × 0.125 mm	Not available			N/A	N/A			N/A
Suphanantachat et al.2017 [94]	25 patients	Intraoral radiograph (Kodak) with 70 kV, 7 mA, exposure time 0.2–0.4 s | CBCT 3D Accuitomo with volumes of 100 3 100 mm, 80 kV, 5 mA, exposure time of 17.5 s and a voxel size of 0.25 mm	Not available			N/A				N/A
Padmanabhan et al. 2017 [95]	14 patients (20–60 years) with 25 mandibular molar furcation sites	Intraoral periapical radiography | CBCT with 84 kv, 5 mA, 20 s, Voxel size of 180 μm	Intrasurgi-cal measurem-ents							
Zhang et al. 2018 [96]	83 patients with chronic periodontitis	CBCT with a field of view (FOV) of 150 × 90 mm2. The scans were acquired at 90 kVp, 10 mA, 16 s and a 0.2 mm3 voxel size with a Kodak 9500 unit (Carestream Health, Inc., Rochester, NY, USA), Intraoral using the unit (Instrumentarium Dental, Charlotte, NC, USA) operating at 70 kVp, 7 mA , and an exposure time corresponding to the exposed area, Clinical measurements	Not available			N/A			N/A	

Good Quality  Not clear **N/A** Not applicable

**Table 4:** CASP checklist for critical appraisal of randomized clinical trials studies

Criteria	Dutra et al. 2017 [82]	Gupta SJ et al. 2014 [72]	Grimrad et al.2009 [68]	Khosropana et al.2015 [91]	Nemoto et al. 2018 [85]	Bhavsar et al. 2016 [77]	Pajnigara et al. 2017 [79]
Did the trial address a clearly focused issue?	Yes	Yes	Yes	Yes	Yes	Yes	Yes
Was the assignment of patients to treatments randomized?	Yes	Yes	Yes	Yes	Yes	Yes	Yes
Were all of the patients who entered the trial properly accounted for at its conclusion?	Yes	Yes	Yes	Yes	Yes	Yes	Yes
Were patients, health workers and study personnel ‘blind’ to treatment?	Can’t tell^a^	Can’t tell^a^	Yes	Yes	Yes	Yes	Yes
Were the groups similar at the start of the trial	Yes	Yes	Yes	Yes	Yes	Yes	Yes
Aside from the experimental intervention, were the groups treated equally?	Yes	Yes	Yes	Yes	Yes	Yes	Yes
How large was the treatment effect?	Satisfactory	Satisfactory	Satisfactory	Satisfactory	Satisfactory	Satisfactory	Satisfactory
How precise was the estimate of the treatment effect?	Acceptable	Acceptable	Acceptable	Accept-able	Acceptable	Acceptable	Acceptable
Can the results be applied to the local population or in your contex	Yes	Yes	Yes	Yes	Yes	Yes	Yes
Were all clinically important outcomes considered?	Yes	Yes	Yes	Yes	Yes	Yes	Yes
Are the benefits worth the harms and costs?	Yes	Yes	Yes	Yes	Yes	Yes	Yes

^**a**^*Can’t tell* cannot tell; criteria in this tool

### Systematic reviews

The remaining six studies [6, 70, 73, 78, 80, 83] were found to be systematic reviews for which the AMSTAR-2 assessment tool [97] was used. It is a popular instrument modified from the original AMSTAR, which contains 16 checklist questions. (Refer to Table 5). The two authors meticulously screened each study in order to give a suitable answer for each checklist question.

**Table 5:** AMSTAR2 checklist for systematic review appraisal

Criteria	Systematic Reviews					
	Haas et al. 2018 [78]	Anter et al. 2016 [73]	Walter et al. 2016 [70]	Nikolic-Jakoba et al. 2016 [6]	Choi et al. 2018 [80]	Woelber et al. 2018 [83]
1. Did the research questions and inclusion criteria for the review include the components of PICO?	Yes	No	Yes	Yes	Yes	Yes
2. Did the report of the review contain an explicit statement that the review methods were established prior to the conduct of the review and did the report justify any significant deviations from the protocol?	Yes	Yes	Partial yes	Yes	Partial yes	Yes
3. Did the review authors explain their reasons for selection of the study designs for inclusion in the review?	Yes	Yes	Yes	Yes	No “Some failed to continue”	Yes
4. Did the review authors use a comprehensive literature search strategy?	Partial yes	Partial yes	No	Yes	Yes	Yes
5. Did the review authors perform study selection in duplicate?	Yes	Yes	No	Yes	Yes	Yes
6. Did the review authors perform data extraction in duplicate?	Yes	No	Yes	Yes	Yes	Yes
7. Did the review authors provide a list of excluded studies and justify the exclusions?	Yes	Yes	Yes	Partial yes	Partial yes	Yes
8. Did the review authors describe the included studies in adequate detail?	Yes	Partial yes	Partial yes	Yes	Yes	Yes
9. Did the review authors use a satisfactory technique for assessing the risk of bias (RoB) in individual studies included in the review?	Yes	Partial yes	No	Yes	No	No
10. Did the review authors report on the sources of funding for the studies included in the review?	No	No	No	No	No	No
11. If meta-analysis was performed, did the review authors use appropriate methods for a statistical combination of results?	Yes	No meta-analysis conducted	No meta-analysis	No meta-analysis conduct-ed	No meta-analysis	No meta-analysis performed
12. If meta-analysis was performed, did the review authors assess the potential impact of RoB on individual studies based on the results of the meta-analysis or other evidence synthesis?	No	No meta-analysis conducted	No meta-analysis	No meta-analysis conducted	No meta-analysis performed	No meta-analysis performed
13. Did the review authors account for RoB in individual studies when interpreting/ discussing the results of the review?	Yes	Yes	No^a^ “No RoB assessed”	Yes	No	No
14. Did the review authors provide a satisfactory explanation for, and discussion of, any heterogeneity observed in the results of the review?	Yes	Yes	Yes	Yes	Yes	Yes
15. If they performed quantitative synthesis, did the review authors carry out an adequate investigation of publication bias (small study bias) and discuss its likely impact on the results of the review?	No	No meta-analy-sis cond-ucted	No meta-analysis conduct-ed	No meta-analysis conduc-ted	No meta-analysis performed	No meta-analysis perform-ed
16. Did the review authors report any potential sources of conflict of interest, including any funding they received for conducting	No	Yes	No	Yes	No	Yes

^a^*No RoB* no risk of bias assessed

## Discussion

Alveolar bone loss is considered a primary symptom of periodontal diseases. Mostly, the assessment and treatment decisions depend on clinical measurements supported by conventional imaging modalities. However, 2D imaging has its own limitations for detecting bone defects, including overlapping. An estimation of bone loss bucco-lingually has led to the consideration of 3D imaging. However, to what extent the CBCT is effective in the diagnosis of periodontal diseases is not yet clear. Accordingly, our systematic review was designed to summarize the available evidence according to the hierarchal model of efficacy developed by Fryback et al. [33].

In our systematic review, we decided to exclude studies that are published in any language other than English because of time restriction. In addition, case reports and narrative reviews are considered in the literature as low-evidence studies. Studies addressing periapical conditions and implant-related periodontal problems were also excluded as they are beyond our aspect in this review. In the meantime, it was decided to not include studies conducted ex vivo where the periodontal defects are created artificially since we believe those results will not mimic the CBCT’s performance when conducted on humans.

### Technical efficacy level

It seems most of the studies conducted on the use of CBCT in periodontal disease were aimed at performance detection, accuracy estimation, or the treatment outcome assessment. The authors found no study reported in the literature dealt with the technical aspect of CBCT.

### Diagnostic accuracy level

As mentioned earlier in this review, the QUADAS 2 tool was used for the quality assessment of diagnostic accuracy studies. Only studies conducted in vivo were included in this review. Some studies did not use explicit reference standards to compare CBCT with other modalities [71, 89, 90, 93, 94].

Cimbaljevic et al. [63] compared the periodontal probing with CBCT in the terms of furcation involvement in the absence of a reference standard. Likewise, Darby et al. [64] addressed the discrepancies in the clinical measurements obtained from patients’ records with their available CBCT images. A study conducted by Suphaanantachat et al. [92] compared CBCT to conventional intraoral radiography. However, they did not use an actual reference standard for comparison. Similarly, Zhu J. et al. [86] has focused on the reproducibility of the different parameters of CBCT for the furcation involvement evaluation, and hence, no reference standard was used.

### Diagnostic thinking

A study published by Walter et al. [66] on decision-making revealed discrepancies between clinically and CBCT-based therapeutic treatment approaches. The discrepancy was found after 59–82% of the teeth were investigated to find out whether less invasive or most invasive treatment should be considered. However, they concluded that CBCT provides informative details in cases of furcation involvement, and hence, it is considered a reliable tool in decision-making regarding treatment of furcation involvement.

### Therapeutic efficacy

According to our interpretation and in correlation with the hierarchical model of efficacy [33], we found that the study conducted by Pajnigara et al. [67] fits on this level. They investigated the pre and post-surgical measurements of clinical and CBCT for furcation defects. Although they reported statistically significant differences between; clinical-presurgery CBCT (*P* < 0.0001, 95% CI) and clinical-post surgery CBCT; the three-dimensional imaging gives dental practitioners the chance to optimize treatment decisions and assess the degree of healing more effectively.

### Patient’s outcome efficacy

Our systematic review has revealed eight studies that used CBCT to assess the results of treatment provided for periodontal diseases [68, 72, 75, 77, 79, 82, 85, 98]. However, it seems that this study is in disagreement with a previously published review [6]. They did not identify any study on the level of patient outcome. The reason for this could be the difference between our inclusion and exclusion criteria and theirs. All studies agreed that CBCT is a reliable tool in the assessment of the results of treatment using a bone graft.

### Societal efficacy

The study reported by Walter et al. [69] has shown that the use of CBCT decreases the cost and time for periodontitis screening. However, CBCT should only be advised in cases of advanced therapy. Further studies with a sufficient number of patients were suggested.

### Systematic reviews

Our review has resulted in six studies, which are systematic reviews. Each review is supposed to adhere to the criteria provided by AMSTAR and scores YES whenever applicable. The review published by Haas et al. [78] did not elaborate on whether they included the study registries or consulted content experts in the field in terms of comprehensive literature search strategy. Although a meta-analysis was conducted in such a review, the review authors did not assess the potential impact of risk of bias on the results of the meta-analysis or other evidence synthesis. Moreover, the authors did not carry out an adequate investigation of publication bias (small-study bias) or discuss its likely impact on the results of the review. Based on our interpretation, the study has not reported any source of funding or mentioned any conflict of interest.

The study by Walter et al. [79] did not clearly have an explicit statement that the review methods were established prior to the conduct of the review and did not justify any significant deviations from the protocol. In addition, only one database has been searched for relevant studies. According to the AMSTAR2 criteria, the included studies were not described adequately. The study has not reported on the source of funding for the individual studies included in the review. To our knowledge, the risk of bias has not been elaborated upon in the relevant sites in the review. Moreover, the review authors did not account for the risk of bias in individual studies when interpreting or discussing the results of the review. In addition, the authors have not reported any source of conflict including any funding they received for conducting the review.

The review by Anter et al. addressed the accuracy of the CBCT as a tool for the measurement of alveolar bone loss in periodontal defects. However, the authors did not report that they followed PICO, which is a framework for review question formulation [36]. In terms of a comprehensive search strategy, we saw that this review did not fulfill the criteria regarding study registries and expert consultation in the field. Furthermore, the authors did not conduct the search in duplicate for the purpose of study selection. The review authors had also not performed data extraction in duplicates. According to our interpretation, the included studies were not described in appropriate detail. Additionally, the source of funding for each relevant individual study was not reported.

The study reported by Choi et al. [80] did not specify whether if there was a deviation from protocol, meta-analysis plan, or causes of heterogeneity if appropriate. In addition, a list of the excluded study in association with a justification for exclusion of each potential study has not been provided. Regardless of whether it is one of the targets of the review, this review has not discussed any potential risk of bias of the included studies. Moreover, the source of funding of each included study was also not reported. It could be included that this review does fulfill the AMSTAR2 [97] checklist to some extent.

The review by Woelber et al. [83] neither mentions any deviation from protocol whenever applicable nor elaborates on if is a plan for meta-analysis, if appropriate. In addition, a plan for investigating the possible causes, if appropriate, regarding heterogeneity was also not reported. The source of funding for each included study was not reported either. To some extent, the review fulfills the checklist of AMSTAR2.

According to our systematic review and AMSTAR2 tool, we found the review conducted by Nikolic-Jakoba et al. [6] best fulfills the tool criteria. However, the study’s authors did not justify the reason for exclusion of each potentially relevant study from the review. As other reviews were included in our study, the source of funding of each included publication was not reported.

## Conclusion

We concluded that most of the studies conducted on the rule of CBCT in periodontal diseases were at diagnostic accuracy level followed by the patient outcome level. Accordingly, it was found that CBCT is quite beneficial and accurate in the diagnosis of infra-bony defects and furcation involvement. Similarly, it is reliable in the assessment of the outcome of periodontal surgery and regenerative therapy. Furthermore, more studies with a larger cohort on the level of diagnostic thinking, therapeutic, and societal efficacy are needed to set up a clear guideline and evidence for the usefulness of CBCT.

## Data Availability

The datasets used and/or analyzed during the current study are available from the corresponding author on reasonable request.

## Abbreviations

CBCT: Cone beam computed tomography
CT: Computed tomography
PRISMA: Preferred items for systematic review and meta-analysis
CRD: Center for reviews and dissemination
PICO: Population, or Problem, Intervention or Exposure, Comparison (if appropriate), Outcome you would like to measure or achieve
QUADAS2: Quality assessment tool for diagnostic accuracy studies
CASP: Critical appraisal skills program
AMSTAR: A measurement tool to assess systematic review
2D: Two-dimensional
3D: Three-dimensional
